# Assessing glyphosate in water, marine particulate matter, and sediments in the Lagoon of Venice

**DOI:** 10.1007/s11356-021-16957-x

**Published:** 2021-10-14

**Authors:** Matteo Feltracco, Elena Barbaro, Elisa Morabito, Roberta Zangrando, Rossano Piazza, Carlo Barbante, Andrea Gambaro

**Affiliations:** 1 Institute of Polar Sciences, National Research Council (CNR-ISP), Via Torino, 155 - 30172 Venice Mestre, VE Italy; 2 Department of Environmental Sciences, Informatics and Statistics, Ca’ Foscari University of Venice, Via Torino, 155 - 30172 Venice Mestre, VE Italy

**Keywords:** Glyphosate, HPAEC-MS/MS, Water, Suspended particulate matter, Sediment, Venice Lagoon

## Abstract

Lagoon water, suspended particulate matter, and sediment samples from seven sites at Lagoon of Venice were collected from 2019 to 2021 in order to study the presence of the herbicide glyphosate (*N*-(phosphonomethyl)glycine), among the most widely used agricultural chemicals worldwide, but its occurrence in lagoon water environment has not been deeply investigated. The sites were selected considering a supposed diversity of inputs and of pollution levels. An analytical method based on ion chromatography coupled with tandem mass spectrometry was optimized and validated for lagoon water, marine particulate matter, and sediment samples. Maximum concentrations of glyphosate were 260 and 7 ng L^−1^ for lagoon water and suspended particulate matter, respectively, and 15 ng g^−1^ for sediment, with some spatial and temporal fluctuations. Our results demonstrate that glyphosate content in the Venice Lagoon mainly depends on external forcing from river inlets and agricultural lagoon activities.

**Supplementary Information:**

The online version contains supplementary material available at 10.1007/s11356-021-16957-x.

## Introduction

Glyphosate (*N*-(phosphonomethyl)glycine) is a broad-spectrum herbicide used to control a wide range of pesticides. Glyphosate was introduced under the industrial name of Roundup® in 1974. Its extensive use is due to several characteristics: broad spectrum, high efficacy, and low cost. These characteristics have expanded its use representing the largest portion (~ 12% in 2012) of the global herbicide market. This herbicide is used mainly in conventional crops, which represent about 55% of total crop volume in 2015. Among the users, the Asian countries consume most of the product (31%), followed by Latin America (20%) and Europe (16%). Due to its efficiency, it is also used for the urban areas weeding, public parking, and for the maintenance of roads, motorways, large industrial sites, railway networks, etc. For the Veneto region in 2015, 446 tons of active ingredients were sold (ARPAV—monitoring of glyphosate, AMPA, and ammonium glufosinate in the surface waters of the Veneto, see: arpa.veneto.it/temi-ambientali/acqua/file-e-allegati/documenti/acque-interne/acque-superficiali). The Veneto region has reached the use of 20 k tons in 2018 for pesticides, due to the exponential expansion of vineyards since 2010. Furthermore, Veneto has been the major phytosanitary-consumer region of Northern Italy (see: dati.istat.it).

Glyphosate is commonly used as isopropylamine salt. It is a polar water-soluble organic compound that makes complexes easily. The degradation pathway of glyphosate through laboratory experiments is well studied and is mainly determined by microbial processes (Carlisle and Trevors 1988; Gimsing et al. 2004; Mercurio et al. 2014, 2015; Rodríguez-Gil et al. 2021). Considering field conditions, glyphosate is degraded to aminomethylphosphonic acid (AMPA) and CO_2_. From literature studies, glyphosate’s half-life in surface waters and soil ranges from 2 to 91 days and from 2 to 215 days, respectively (Battaglin et al. 2014; Castro Berman et al. 2018). AMPA has a longer half-life in soil (from 60 to 240 days).

In the last decades, glyphosate was considered having a good ecotoxicological profile; however, concerns about its carcinogenicity was pointed out by the international scientific community (Williams et al. 2000; Ighalo et al. 2021). In 2017, after the lack of a decision to ban or extend glyphosate after several discussions, a qualified majority of European Member States voted to allow the use of glyphosate for five more years with some specific provisions (DeSesso et al. 2017). Apart from the unsettled question of carcinogenicity, there is growing evidence on chronic and acute toxic effects of glyphosate on different organisms (i.e., algae, amphibians, fish, birds) (Kittle and McDermid 2016; De María et al. 2021; Du-Carrée et al. 2021). Tadpoles chronically exposed to glyphosate were smaller and slower to transform into frogs, although this observation occurred following glyphosate exposure concentrations ranging from 0.6 to 18 mg L^−1^ (Howe et al. 2004). Several methods have been explored to remove glyphosate from the water systems. Among these methods, adsorption has been most interesting due to its low cost, environmentally friendly operation, and availability of material of adsorbents (Ighalo et al. 2021 and references therein).

Glyphosate and AMPA have commonly been mainly detected in surface water in all European countries, with a wide range of concentration levels (Skeff et al. 2015, 2018; Grandcoin et al. 2017; Poiger et al. 2017; Richmond 2018). After entering the seawater, pesticides with a strong adsorption propensity can adsorb to the suspended particulate matter before settling on sediments (Doong et al. 2002). However, few studies have investigated the behaviour of highly polar compounds like glyphosate (pK_a_ = 0.8–10.14) (Skeff et al. 2018). As a result, a systematic assessment of glyphosate concentrations in lagoon water, suspended particulate matter, and sediments are scarce, and for most parts of the world, non-existent (Mercurio et al. 2014). The Italian Legislative Decree n. 152/2006 set the maximal annual mean value of 0.1 μg L^−1^ in surface water for each pesticide; no levels were set for seawater, suspended particulate matter, or sediments.

**Fig. 1:**
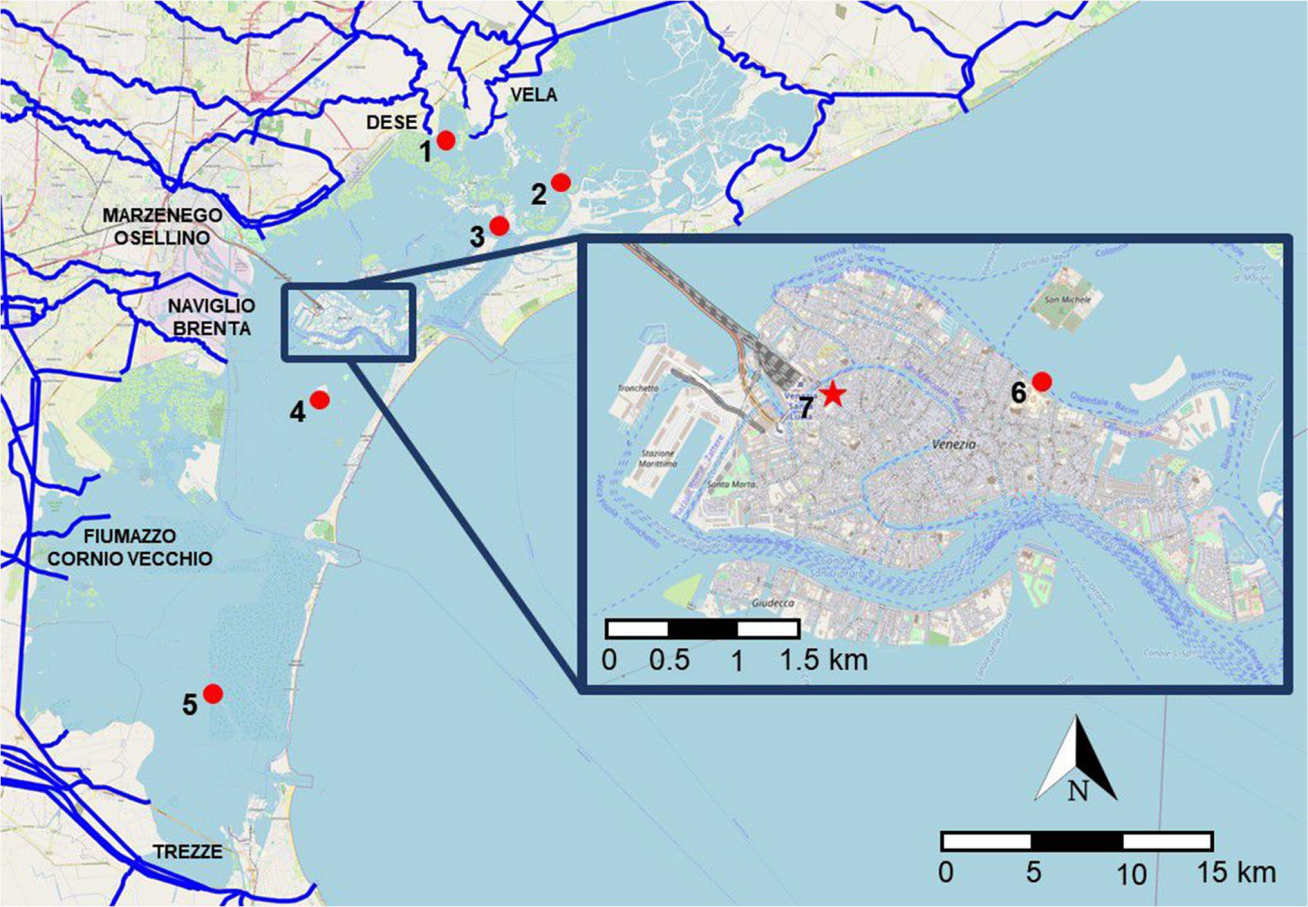
Lagoon water and sediment sampling sites in red including rivers, small streams, and agricultural and industrial drainage ditches across the Lagoon of Venice. (1) Mouth of Dese River, (2) Palude Maggiore, (3) St. Erasmo, (4) Sacca Sessola, (5) Petta di Bo’, (6) Hospital, and (7) Rio Marin

Venice and its lagoon are a UNESCO World Heritage Site since 1987 for the “uniqueness of its cultural values integrated into an extraordinary and outstanding environmental, natural, and landscape context” (UNESCO 1987). The Venice Lagoon is one of the largest lagoons in the Mediterranean Sea (~ 390 km^2^) (Cossarini et al. 2009), and its physics is driven by turbulent and tidal mixing and is influenced by external forcing from ocean and rivers (Dejak et al. 1998). Adriatic seawater exchange occurs through three large entrances (Chioggia, Malamocco, and Lido), but the main active sources of pollutants are the rivers of the watershed; treated and untreated wastewater directly enters the lagoon from the surroundings industrial settlements, agricultural activities, and the Venice historical centre inputs (Suman et al. 2005; Apitz et al. 2007). Glyphosate can reach the lagoon ecosystem directly from the agricultural through the hydrographic network showed in Fig. 1. The quality status of the Venice Lagoon has been studied for several years (Moret et al. 2001; Moret and Gambaro 2005; Morabito et al. 2018; Corami et al. 2020; Pizzini et al. 2021), and some investigations have been done to define the presence of terbuthylazine, which replaced atrazine as a generic herbicide in the early 2000s (Collavini et al. 2005), but no investigations about the presence and seasonal variability have been done for glyphosate so far.

Given the widespread use of glyphosate and doubts regarding its environmental fate and potential effects in aquatic ecosystems and considering the strong influence of river discharges in the lagoon, the present paper aims to develop the analytical method to determine glyphosate, AMPA, and glufosinate in representative samples of dissolved, particulate, and sedimentary phases in the Venice Lagoon from spring 2019 to winter 2021, as well as to assess potential spatial distributions and seasonal trends in concentrations.

## Experimental section

### Reagents and standard solutions

Ultra-grade methanol (MeOH) was purchased from VWR® (Radnor, PA, USA), Ultrapure water (18.2 MΩ, 1 ppb TOC) was produced using a Purelab Ultra System (Elga®, HighWycombe, UK). Glyphosate (s), AMPA (s), glufosinate (s), and the internal standards glyphosate-2-^13^C,^15^ N (s) and AMPA-^13^C,^15^ N (liquid, 100 μg mL^−1^ in H_2_O) were purchased from Merck KGaA (Darmstadt, Germany). The solid standards (purity > 99%) were diluted in ultrapure water.

### HPAEC-MS/MS

Determination and quantification of pesticides were performed using an ion chromatograph (Dionex™, Thermo Scientific™, ICS-5000, Waltham, USA) coupled to a Quattro Ultima quadrupole-hexapole-quadrupole (QHQ) mass spectrometer (Waters Corporation, Milford, MA, USA) using a z-spray electrospray source (ESI) that operated in negative mode. Chromatographic separation was carried out using an anion exchange column AS18 2 × 250 mm (Dionex™ ICS 5000 EG, Thermo Scientific™). The gradient of sodium hydroxide (NaOH), produced by an eluent generator, with a 0.25-mL min^−1^ flow rate was 0–2 min gradient 15 mM, 2–9 min gradient from 15 to 60 mM, 9–20 min isocratic step at 100 mM, and 20–35 min, equilibration with 15 mM. The mass spectrometer’s parameters were set as follows: desolvation temperature 450 °C, source temperature 115 °C, ion spray voltage − 2 kV, RF1 2.5, RF2 0.5, and aperture 0.5. Data were collected with multiple reaction monitoring (MRM) mode. The first quadrupole (Q1) selected the molecular ion [M-H]^−^, while the third quadrupole (Q3) selected the product ion. Both Q1 and Q3 were set at unit resolution with a peak width of 0.7 ± 0.1 amu at 50% of maximum peak height. To improve the sensitivity, the cone and collision voltage were set, using direct infusions of 1 mg L^−1^ of each standard. The monitored transition and instrumental parameters for each compound are shown in Table S1. MassLynx V4.1 (Waters Corporation) was used for the identification and quantification of pesticides.

### Sample collection

Lagoon water and sediment samples were collected in 2019, 2020, and early 2021, from seven different stations distributed along the Lagoon of Venice (Fig. 1) at different time intervals. Sampling sites were chosen both to investigate specific emission sources (Mouth of Dese River, St. Erasmo, Venice hospital and Rio Marin) and to obtain an overview of the lagoon area (Palude Maggiore, Sacca Sessola, and Petta di Bò, located in the northern, central, and southern lagoon, respectively). Mouth of Dese River is a sampling site that provides an important input from inland cultivations, while most of the St. Erasmo Island is used for agricultural purposes. The sampling at Rio Marin was only performed in the third and fourth campaigns and, together with Venice Hospital, provide information about the impact of gardening and agriculture activity from Venice historical centre. About 20 L of water were collected at each site by immersing decontaminated steel bottles to a depth of 20 cm below the surface. A 10-cm layer of sediment was sampled by using a Van Veen grab. The sediment samples were homogenized and kept frozen in aluminium containers at − 20 °C until processing and analysis. Sampling site parameters are reported in Table S2. Each sample preparation phase was performed inside an ISO 7 cleanroom at the Ca’ Foscari University of Venice, and all glassware and tools were previously pre-cleaned with dichloromethane and methanol.

### Sample treatment

After sampling, water samples were immediately filtered in the lab using previously calcinated 0.7-μm glass microfiber filters (Sartorius, Göttingen, Germany) for the determination of particulate fraction. Depending on the variable suspended particulate matter load, 0.25 to 1 L of lagoon water were filtered in each filter. To determine and quantify pesticides, the water samples were loaded on a SPE Oasis® HLB cartridge (Hydrophilic-Lipophilic Balance; 6 mL, 200 mg, 30 μm; Waters Corp., Milford, MA, USA), previously conditioned with 10 mL of methanol, followed by 10 mL of ultra-pure water. This step is performed to remove potential matrix-related interfering substances. All the samples were prepared as follows: for lagoon water (LW), samples were twofold diluted with ultrapure water and purified using an IC-AG cartridge (Thermo Scientific®), pre-activated with 2 mL of ultrapure water, to minimize the presence of Cl^−^ and to avoid the contamination of the analytical system. For suspended particulate matter (SPM), the particulate matter collected on a quartz fibre filter was broken up into small pieces and placed in a 15-mL vial (previously cleaned with ultra-pure water by sonication at 25 °C) with steel tweezers. Five millilitres of ultrapure water was added to the substrate before cold-ultrasonically extracting at 10 °C to avoid the possible degradation or/and oxidation of the analytes. The extract has been filtrated through a 0.45-µm PTFE filter (Labware®, Meckenheim, Germany) to remove particulate and filter traces before analysis. For sediment (SED), about 1 g of sediment was cold-ultrasonically extracted with 10 mL of ultrapure water and then filtrated through a 0.45-µm PTFE filter. AMPA and glufosinate in the LW, SPM, and SED were strongly suppressed by the presence of salts, despite the use of IC-AG cartridge, and for this reason, only the presence of glyphosate is here proposed. LW, suspended SPM, and SED samples of Palude Maggiore collected on 20 July 2020 (PM3) were taken as blank because in the preliminary analysis, the target pesticides were not detectable in that area. The extracted fraction of PM3 was used to prepare the matrix-related calibration curve.

## Result and discussion

### Quantitative performances

**Fig. 2:**
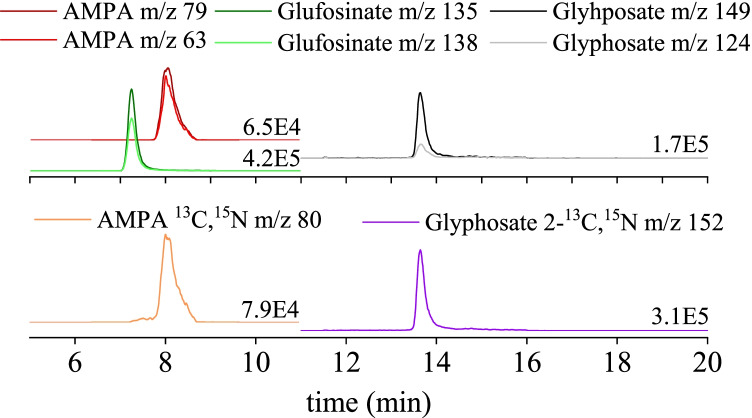
Chromatograms of the target compounds and their internal standard from 5 to 20 min. Both fragments are shown for each compound

Figure 2 shows the chromatographic separations carried out with a Dionex Ion AS18 column of glyphosate, AMPA, glufosinate, and internal standards using the developed chromatographic method. The analytical procedure was validated through the linear range, instrumental limit of detection (LOD), instrumental limit of quantification (LOQ), procedural blank, method detection limit (MDL), method quantification limit (MQL), repeatability, trueness, and extraction yield. The internal standard method was used to quantify the pesticides and labelled glyphosate and AMPA were chosen as internal standards due to the similar instrumental and preanalytical behaviour to target pesticides. The linearity of the calibration curves was evaluated using standard solutions prepared in ultrapure water with a constant concentration of labelled glyphosate and AMPA of 1.26 and 1.00 μg L^−1^, respectively. Linearity was evaluated at 0.025, 0.05, 0.1, 0.2, and 0.5 μg L^−1^ for LW; 0.025, 0.05, 0.1, 0.2, 0.5, and 1 μg L^−1^ for SPM; and 0.2, 0.5, 1, 5, and 10 μg L^−1^ for SED using PM3 samples.

The matrix effect (ME) is obtained by separating the signal response of a standard present in the sample extract with the response of a standard prepared in the ultrapure water, expressing the result as a percentage. A value of 100% is obtained when no there is a matrix effect while a value < 100% is indicative of an ionization suppression (Matuszewski et al. 2003). Figure S1 shows the ME at three glyphosate concentrations (0.05, 0.1, and 0.5 µg L^−1^) in LW, SPM, and SED. To the best of our knowledge, this is the first study where a full quantitative analysis of matrix effects affecting glyphosate analysis in any matrix has been carried out. This matrix effect evaluation has highlighted that an external calibration curve in pure solvents is not a reasonable calibration method as instrumental signal suppression is not properly considered.

By considering the ratio between the concentration of pesticides and internal standards and the ratio between the relative peak areas, linearity was evaluated. LOD and LOQ values are calculated as three and ten times the signal-to-noise ratio, respectively, of the known absolute amounts of the analysed target compound in a standard solution. The MDL and the MQL were evaluated as 3 and 10 times the standard deviation of these field blanks, respectively (Bliesner 2006). Linearity, LOD, LOQ, MDL, and MQL for glyphosate, AMPA, and glufosinate are reported in Table 1. The LODs achieved for glyphosate in this study were at least one order of magnitude lower than other methods developed with ion chromatography reported in recent studies (Zhu et al. 1999; Popp et al. 2008; Chiesa et al. 2019; Dovidauskas et al. 2020). This huge step forward improving the sensitivity in terms of LODs allowed us to assess the presence of glyphosate at an ultra-trace level. The instrumental precision was evaluated and CV% value (reported as a percentage and calculated from the average (*A*) and standard deviation (*SD*), calculated as (*SD*/*A*) × 100) was always below 10%.

**Table 1:** Quantitative performances for target compounds in ultrapure water (UPW), lagoon water (LW), suspended particulate matter (SPM), and sediments (SED). LOD, LOQ, MDL, and MQL are shown in ng L^−1^ for UPW, LW, and SPM and in ng g^−1^ for SED

	UPW (ng L^−1^)			LW (ng L^−1^)	SPM (ng L^−1^)	SED (ng g^−1^)
	Glyphosate	AMPA	Glufosinate	Glyphosate	Glyphosate	Glyphosate
*R*^2^	0.9983	0.9995	0.9981	0.9989	0.9992	0.9994
LOD	5.4 ± 0.5	1.6 ± 0.1	1.4 ± 0.2	12 ± 1	0.06 ± 0.01	0.12 ± 0.02
LOQ	18 ± 2	5.3 ± 0.5	4.6 ± 0.2	40 ± 4	0.20 ± 0.02	0.4 ± 0.1
MDL	0.2	0.1	0.1	2	2	0.2
MQL	0.7	0.6	0.6	7	8	0.7
			Extraction y. (%)	/	93	94
			Error (%)	− 3	− 2	− 1

Another important parameter of the method validation is trueness. It is expressed as a per cent error (Table 1), calculated as (*Q* − *T*)/*T* × 100 where *Q* is the determined value and *T* is the “true” value. The error for glyphosate was calculated performing the same pre-analytical procedure achieved with the samples from the Lagoon of Venice. For the evaluation of the extraction yield to estimate the procedural extraction efficiency for SPM and SED, the isotopically labelled glyphosate-2-^13^C,^15^ N was added after the PTFE filtration. Extraction yield and error values for glyphosate in SPM and SED are reported in Table 1.

The recovery of the analytical procedure for SPM and SED for glyphosate ranged between 93 and 94%, comparable to the values found by Peruzzo et al. (2008) and Imfeld et al. (2013) that obtained a recovery of 86% in soybean cultivation and wetland sediments, respectively. The method allows the analysis of several samples, which is particularly interesting when a routinely monitor the seawater quality of a region is needed.

### Occurrence of glyphosate in the Venice Lagoon

Glyphosate was determined in the LW, SPM, and SED collected in the Lagoon of Venice in spring 2019, autumn 2019, summer 2020, and winter 2021 (Table S2) to assess the occurrence of glyphosate depending on the seasons and to understand how the rivers and/or the local inputs can influence its concentration in the Venice Lagoon. Yearly, the Regional Agency for the Environmental Prevention and the Protection of Veneto (ARPA Veneto) presents a report of the water quality of the Veneto Region, studying plenty of environmental parameters and pollutants. From 2014 to 2019, ARPA Veneto has monitored the presence of glyphosate in some rivers that flows into the Lagoon of Venice: driven by the growing diversity of uses and dramatic increases in volumes applied, the rivers Dese, Zero, Osellino, and Naviglio Brenta (Fig. 1) showed a non-negligible presence of glyphosate in 2018 and 2019, with a constant exceeding of the concentration limits fixed by the Italian Legislative Decree n. 152/2006 (0.1 µg L^−1^).

The Osellino and Dese rivers had the annual highest concentration in 2019, reaching 0.4 and 0.3 µg L^−1^, respectively. No data are present about the seasonal variability of pesticides in these rivers. The occurrence of polar pesticides as glyphosate in the Lagoon of Venice has not been established: considering the strong influence of river discharges in the lagoon, the possible impact on the microbial community (Ker et al. 2006; Gaupp-berghausen et al. 2015) and the effects of herbicides on non-target aquatic species (Kittle and McDermid 2016) are crucial to identify the amount of glyphosate in the Venice Lagoon environment.

**Fig. 3:**
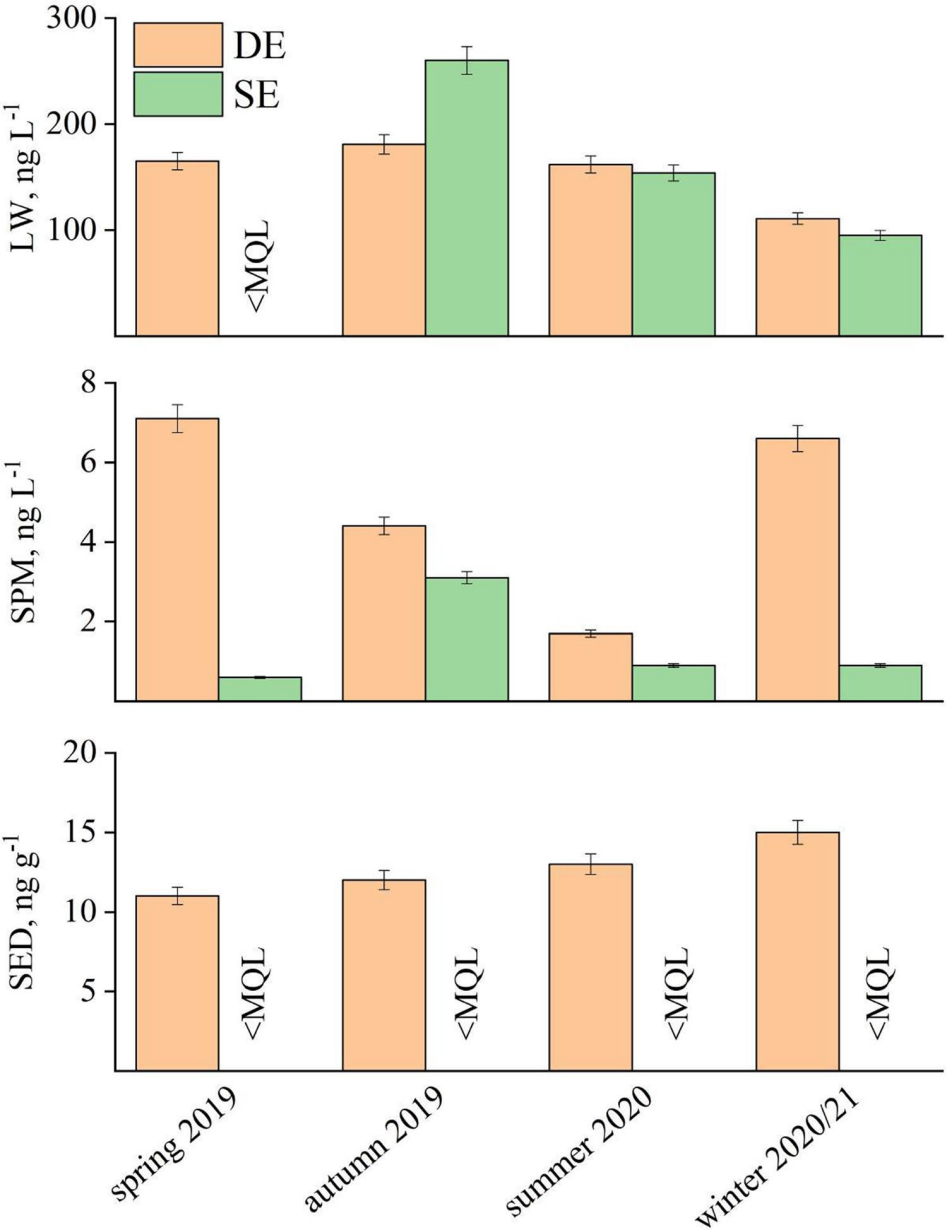
Seasonal variation of glyphosate in Mouth of Dese River (DE) and St. Erasmo (SE)

The glyphosate concentrations for each season in LW, SPM, and SED, in the sites St. Erasmo and Mouth of Dese River, are provided in Fig. 3. The concentrations of glyphosate during the whole sampling period are reported in Table S3. Our results showed the presence of glyphosate mainly in St. Erasmo (SE) and Mouth of Dese River (DE) suggesting that the local and river intake of glyphosate was critical, either from agricultural or non-agricultural activities. Considering the ARPA Veneto values, the presence of glyphosate in the DE could be attributed to agricultural activities, but also to point sources, such as the cleaning of machinery on the riverbank, discarding of recipients with herbicide wastes. Glyphosate can be exported mainly by runoff and by underground leaching from agricultural soils towards surface water, especially when rainfall occurs close to its application (Yang et al. 2015; Grandcoin et al. 2017). The occurrence of glyphosate in the St. Erasmo is likely due to the agricultural activities and vineyards present throughout the island. Apart from DE and SE, the glyphosate concentrations in the other sampling sites were below the MQL. Domestic and urban usage and tidal circulation could explain the sporadic presence of glyphosate in SPM and SED in Rio Marin and Petta di Bò sites (Table S3).

The glyphosate loads in the SPM and SED were always far greater in DE, compared to SE. On the other hand, glyphosate in St. Erasmo LW was < BDL in spring 2019 and showed a similar concentration and trend compared to Dese for the rest of the sampling (Fig. 3). This is might due to the glyphosate adsorbing properties to soil particles (Giesy et al. 2000): during transport over long distances from inland cultivation by Dese River, glyphosate is able to interact with water-soluble organic matter, clay particles, and iron oxides which also belong to the colloidal fraction (Vereecken 2005). This leads to a water-to-colloid transfer during the glyphosate transport. This phenomenon in SE seems to have a much smaller effect compared with DE. This could be explained by the constant tidal removal process of dissolved glyphosate by Adriatic seawater exchange that lacks the possibility to undergo the colloid transfer. The absence of glyphosate in sediments of SE site is may due to the grave seabed that does not favour the adsorption of glyphosate.

The reported seasonal loads represent the budget of glyphosate received by the Venice Lagoon from the drainage basin and are therefore fundamental in planning for the control of water quality. In the framework of system management, an interesting result is related to the temporal variability of the delivered glyphosate and to the importance of regional rainfall events on the overall pollutant transfer (Collavini et al. 2005). Consequently, the seasonal variability is particularly visible for DE in all the studied matrixes (Fig. 3). The risk of offsite transport glyphosate should be particularly evaluated due to the features of the Dese River. The river is a resurgence stream and flows in a predominantly agrarian territory, with intensive farming activities (89%). A more limited portion of the watercourse (11%) runs through urbanized areas. The results shown in Fig. 3 revealed that the concentration trend of glyphosate in the Mouth of Dese River site is strongly dependent on the matrix. LW showed the highest concentration in summer 2020 for both sites, following seasonal-related behaviour connected to the spraying of herbicides during seasonal agriculture. While the load of glyphosate on SED was quite constant through the sampling campaign in the DE site, SPM show an enrichment in spring 2019 and winter 2020/2021. The mechanism behind any seasonality in the Venice Lagoon of glyphosate at the DE can only be hypothesized. As described above and in other studies (Ronco et al. 2016; Mac Loughlin et al. 2020), the presence of glyphosate is very often associated with the SPM and its trend can be associated to local rainfall and water-to-colloid transfer during transport. Furthermore, it is demonstrated that pH, salinity, and temperature variability significantly influence the adsorption behaviours of glyphosate (Zhang and Huang 2011) and therefore its concentration in the LW. A decrease in either the seawater pH or the temperature enhances the adsorption of these compounds onto marine sediments, while the negative correlation between salinity and adsorption suggests the greater mobility of these compounds in marine than in freshwater systems (Skeff et al. 2018). Further investigation is required to link the evolution of dissolved and particulate pesticides, in order to better understand the relationship between them and the effect of their presence in the Venice Lagoon.

## Conclusions

The impacts of deposition and resuspension of herbicides on environment water bodies, aquatic lives, and plants are not fully understood. The literature information about hazard assessment for glyphosate shows that both aquatic and benthic organisms of some areas of the Venice Lagoon may be at risk in when intensive herbicide usage are applied. In this study, a method for the quantification in the marine environment of glyphosate, using a HPAEC-MS/MS system, was developed. We obtained a sensitive method with instrumental detection limits of 12 ng L^−1^, 0.06 ng L^−1^, and 0.12 ng g^−1^ for LW, SPM, and SED respectively. To our knowledge, this is the first study reporting the occurrence of glyphosate in the water, suspended particulate matter, and sediment of Venice Lagoon. A noteworthy accomplishment in this study was to highlight the impact of herbicides on this environment. In several sites, the concentrations were below of concentration limits fixed by the Italian Legislative Decree n. 152/2006 (0.1 µg L^−1^) for freshwater, although relevant concentrations were found in two sites linked with agricultural practices. Considering the major input of glyphosate from river inlets, the presence of this herbicide suggests a non-negligible influence on northeast Italian agricultural activities and vineyards. Further investigation is also necessary, because glyphosate is still not generally considered in most marine monitoring activity despite its wide use due to the massive presence of vineyards in Northern Italy.

## Supplementary Information

Below is the link to the electronic supplementary material.

Supplementary file1 (DOCX 88 KB)

## Data Availability

Not applicable.
