# Supplementary material for: Assessing glyphosate in water, marine particulate matter, and sediments in the Lagoon of Venice
Source: Environ Sci Pollut Res Int. 2021 Oct 14;29(11):16383–91. doi: 10.1007/s11356-021-16957-x (PMC8827352; doi:10.1007/s11356-021-16957-x)
Supplement: Supplementary file 1 — Supplementary file1 (DOCX 88 KB) [file 11356_2021_16957_MOESM1_ESM.docx]

**Assessing glyphosate in water, marine particulate matter and sediments in the Lagoon of Venice**

Matteo Feltracco^a,b^, Elena Barbaro^a,b^, Elisa Morabito^b^, Roberta Zangrando^a,b^, Carlo Barbante^a,b^, Andrea Gambaro^b,a^

^a^ Institute of Polar Sciences, National Research Council (CNR-ISP), Via Torino, 155 - 30172 Venice Mestre (VE), Italy

^b^ Department of Environmental Sciences, Informatics and Statistics, Ca’ Foscari University of Venice, Via Torino, 155 - 30172 Venice Mestre (VE), Italy

Corresponding author: Matteo Feltracco - Institute of Polar Sciences, National Research Council (CNR-ISP), Via Torino, 155 - 30172 Venice Mestre (VE), Italy

**Supporting information**

**Tables – 2**

**Figures – 3**

**Table S1.** Elemental composition, precursor ion (m/z), fragment (m/z), cone and collision voltage.

| Compound | Elemental composition | [M-H^-^] | Fragments | Cone (V) | Collision (V) |
| --- | --- | --- | --- | --- | --- |
| Glyphosate | C_3_H_8_NO_5_P | 168 | 149>124 | 50, 48 | 9, 11 |
| AMPA | CH_6_NO_3_P | 110 | 79>63 | 49, 46 | 18, 17 |
| Glufosinate | C_5_H_12_NO_4_P | 180 | 135>118 | 46, 48 | 15, 17 |
| Glyphosate-2-^13^C,^15^N | C_3_H_8_NO_5_P | 170 | 152>126 | 50, 46 | 10, 11 |
| AMPA-^13^C,^15^N,D_2_ | CH_6_NO_3_P | 112 | 80>63 | 49, 43 | 12, 14 |

**Table S2.** Sampling data.

| Spring 2019 | Latitude N | Longitude E | Date | Time | T (°C) | pH | Eh (mV) |
| --- | --- | --- | --- | --- | --- | --- | --- |
| Petta di Bò (PB1) | 45°16’1.65’’ | 12°15’0.32’’ | 02/04/2019 | 12:30 | 16.2 | 8.5 | 233 |
| Palude Maggiore (PM1) | 45°29’37.47’’ | 12°28’44.00’’ | 16/05/2019 | 12:00 | N/A | N/A | N/A |
| Sacca Sessola (SS1) | 45°24’9.137’’ | 12°19’10.095’’ | 02/04/2019 | 11:15 | 15.8 | 8.2 | 217 |
| Hospital (OS1) | 45°26’27.46’’ | 12°20’41.892’’ | 18/04/2019 | 14:00 | N/A | N/A | N/A |
| Mouth of Dese River (DE1) | 45°31’15.04’’ | 12°23’14.30’’ | 16/05/2019 | 10:30 | N/A | N/A | N/A |
| S. Erasmo (SE1) | 45°28’22.12’’ | 12°25’42.45’’ | 16/05/2019 | 13:00 | N/A | N/A | N/A |
| Autumn 2019 |  |  |  |  |  |  |  |
| Petta di Bò (PB2) | 45°16’1.65’’ | 12°15’0.32’’ | 24/10/2019 | 11:30 | 20.0 | 8.18 | 192 |
| Palude Maggiore (PM2) | 45°29’37.47’’ | 12°28’44.00’’ | 07/11/2019 | 12:20 | 16.2 | 8.48 | 146.9 |
| Sacca Sessola (SS2) | 45°24’9.137’’ | 12°19’10.095’’ | 24/10/2019 | 12:40 | 20.4 | 8.20 | 186 |
| Hospital (OS2) | 45°26’27.46’’ | 12°20’41.892’’ | 23/10/2019 | 15:00 | 19.4 | 8.27 | 120.4 |
| Mouth of Dese River (DE2) | 45°31’15.04’’ | 12°23’14.30’’ | 07/11/2019 | 11:20 | 13.4 | 8.00 | 176.6 |
| S. Erasmo (SE2) | 45°28’22.12’’ | 12°25’42.45’’ | 07/11/2019 | 13:20 | 15.1 | 7.98 | 56.0 |
| Summer 2020 |  |  |  |  |  |  |  |
| Petta di Bò (PB3) | 45°16’1.65’’ | 12°15’0.32’’ | 13/07/2020 | 11:30 | 24.6 | 8.25 | 136 |
| Palude Maggiore (PM3) | 45°29’37.47’’ | 12°28’44.00’’ | 20/07/2020 | 13:00 | 26.5 | 8.42 | 127 |
| Sacca Sessola (SS3) | 45°24’9.137’’ | 12°19’10.095’’ | 13/07/2020 | 13:30 | 25.0 | 8.53 | 158 |
| Hospital (OS3) | 45°26’27.46’’ | 12°20’41.892’’ | 08/07/2020 | 12:55 | 26.0 | 8.05 | 118 |
| Mouth of Dese River (DE3) | 45°31’15.04’’ | 12°23’14.30’’ | 20/07/2020 | 14:20 | 25.0 | 8.33 | 120 |
| S. Erasmo (SE3) | 45°28’22.12’’ | 12°25’42.45’’ | 08/07/2020 | 11:53 | 28.0 | 8.36 | N/A |
| Rio Marin (RM3) | 45°26’20.42 | 12°19’33.23’’ | 21/07/2020 | 14:20 | 27.2 | 8.14 | N/A |
| Winter 2020/2021 |  |  |  |  |  |  |  |
| Petta di Bò (PB4) | 45°16’1.65’’ | 12°15’0.32’’ | 19/01/2021 | 10:45 | 3.4 | 8.08 | 143.8 |
| Palude Maggiore (PM4) | 45°29’37.47’’ | 12°28’44.00’’ | 03/02/2021 | 12:00 | N/A | N/A | N/A |
| Sacca Sessola (SS4) | 45°24’9.137’’ | 12°19’10.095’’ | 19/01/2021 | 13:00 | 3.8 | 8.17 | 159 |
| Hospital (OS4) | 45°26’27.46’’ | 12°20’41.892’’ | 11/02/2021 | 10:00 | N/A | 8.11 | 165 |
| Mouth of Dese River (DE4) | 45°31’15.04’’ | 12°23’14.30’’ | 03/02/2021 | 11:00 | N/A | N/A | N/A |
| S. Erasmo (SE4) | 45°28’22.12’’ | 12°25’42.45’’ | 03/02/2021 | 13:00 | N/A | N/A | N/A |
| Rio Marin (RM4) | 45°26’20.42 | 12°19’33.23’’ | 28/01/2021 | 16:00 | 5.5 | 7.0 | N/A |

**Table S3.** Concentrations of glyphosate in LW, SPM and SED. MQL: Method Quantification Limit.

| Spring 2019 | LW ng L^-1^ | SPM ng L^-1^ | SED ng g^-1^ |
| --- | --- | --- | --- |
| Petta di Bò (PB1) | <MQL | 0.2 | <MQL |
| Palude Maggiore (PM1) | <MQL | <MQL | <MQL |
| Sacca Sessola (SS1) | <MQL | 0.2 | <MQL |
| Hospital (OS1) | <MQL | <MQL | <MQL |
| Mouth of Dese River (DE1) | 165 | 7.1 | 11 |
| S. Erasmo (SE1) | <MQL | 0.6 | <MQL |
| Autumn 2019 |  |  |  |
| Petta di Bò (PB2) | <MQL | 0.2 | <MQL |
| Palude Maggiore (PM2) | <MQL | <MQL | <MQL |
| Sacca Sessola (SS2) | <MQL | <MQL | <MQL |
| Hospital (OS2) | <MQL | <MQL | <MQL |
| Mouth of Dese River (DE2) | 181 | 4.4 | 12 |
| S. Erasmo (SE2) | 260 | 3.1 | <MQL |
| Summer 2020 |  |  |  |
| Petta di Bò (PB3) | <MQL | <MQL | <MQL |
| Palude Maggiore (PM3) | <MQL | <MQL | <MQL |
| Sacca Sessola (SS3) | <MQL | <MQL | <MQL |
| Hospital (OS3) | <MQL | 0.7 | <MQL |
| Mouth of Dese River (DE3) | 162 | 1.7 | 13 |
| S. Erasmo (SE3) | 154 | 0.9 | <MQL |
| Rio Marin (RM3) | <MQL | <MQL | 6 |
| Winter 2020/2021 |  |  |  |
| Petta di Bò (PB4) | <MQL | <MQL | 4 |
| Palude Maggiore (PM4) | <MQL | <MQL | <MQL |
| Sacca Sessola (SS4) | <MQL | <MQL | <MQL |
| Hospital (OS4) | <MQL | <MQL | <MQL |
| Mouth of Dese River (DE4) | 111 | 6.6 | 15 |
| S. Erasmo (SE4) | 95 | 0.9 | <MQL |
| Rio Marin (RM4) | <MQL | <MQL | <MQL |


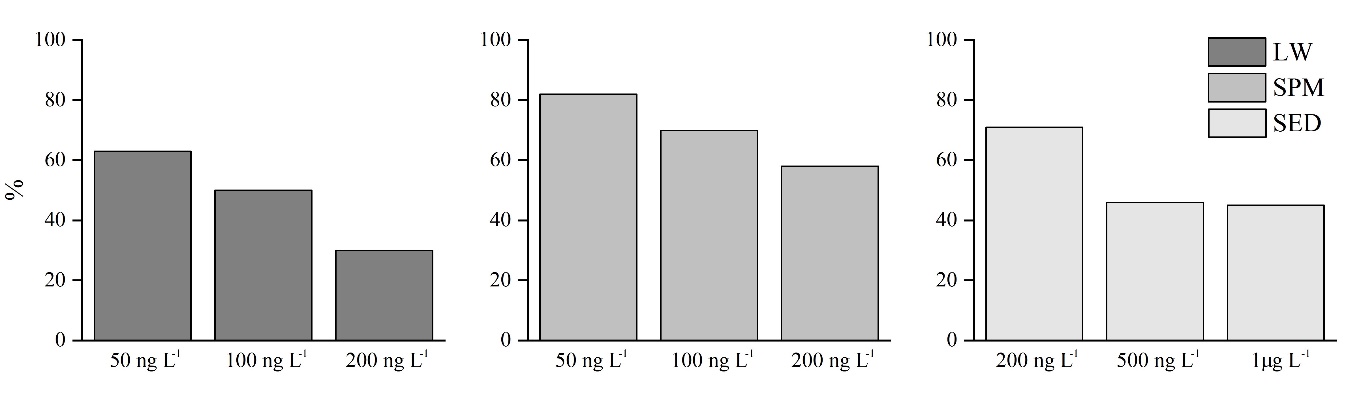


**Figure S1.** A quantitative evaluation of matrix effects in the method for the determination of glyphosate in seawater, suspended particulate matter and sediments.
